# Availability & Applicability of the National Institute of Health Stroke Scale at Time of Imaging Interpretation for Code Stroke in the Community Hospital Setting

**DOI:** 10.5334/jbsr.3932

**Published:** 2025-06-16

**Authors:** Brandon Funk, Behnaz Khazai, Brian Shim, Jacob Van Vorst, Liam du Preez, Cecile Moliva Anendaga, Michael Hollander

**Affiliations:** 1 Norwalk Hospital, USA

**Keywords:** acute ischemic stroke, ischemic stroke, stroke, neuroradiology, neurology, emergency service

## Abstract

*Objective:* Stroke is a leading cause of global disability and mortality, a burden projected to grow as populations age worldwide. Early recognition and our ever-advancing interventions can substantially change outcomes and final costs. This has warranted adapting strategies that maximize sensitivity, including a lower threshold for code stroke imaging. This growing demand requires matching resources, an issue that is particularly noticeable in community hospital settings. The National Institutes of Health Stroke Scale (NIHSS) is a well-established quantitative tool for guiding clinical management. This study aimed to assess the availability and applicability of the NIHSS at the time of imaging for code stroke evaluations in a community hospital setting and in comparison to the previous studies conducted in tertiary academic centers.

*Materials and methods:* We performed a retrospective analysis of all code stroke activations at a community Level 1 stroke center from October 2021 to September 2023, when institutional benchmarks were last adapted. All patients underwent non-contrast head CT, CT angiography of the head and neck, and subsequent brain MRI. Data collected included NIHSS documentation status, door-to-CT and door-to-NIHSS times, imaging positivity, final neurological diagnosis, and therapeutic interventions. Statistical analysis included descriptive statistics, *t*-tests, and receiver operating characteristic (ROC) analysis using NIHSS to predict positive strokes.

*Results:* A total of 291 patients were included (151 women, 140 men). NIHSS documentation prior to imaging was available in 61.2% of cases. Median door-to-CT time was 12 minutes, while median door-to-NIHSS time was 29 minutes. Imaging was positive for acute stroke in 33.6% of cases. Patients with NIHSS documented prior to imaging had a higher stroke positivity rate (36.5% vs 28.0%; odds ratio [OR] 1.45 (95% CI 0.86–2.42). The mean NIHSS among all patients was 6.3, and higher scores correlated with positive imaging (mean 9.1 vs 3.7). ROC analysis for NIHSS predicting imaging positivity yielded an AUC of 0.69. Notably, eight patients (2.7% of all patients) had an NIHSS of 0 but demonstrated acute infarcts on imaging.

*Conclusion:* Our findings demonstrate that NIHSS documentation often lags behind imaging in a community setting, yet its availability can provide useful prognostic information. Higher NIHSS scores correlate with a higher number of positive strokes by imaging, yet, strokes may be seen even with an NIHSS score of 0. These findings emphasize maintaining a low threshold for imaging and the importance of prompt NIHSS documentation, especially in community stroke centers increasingly relying on tele-neurology.

## Introduction

Stroke remains one of the foremost causes of mortality and long-term disability worldwide, contributing substantially to healthcare burdens [1, 2]. Early diagnosis and intervention are crucial to improving outcomes, with imaging playing a central role in differentiating ischemic from hemorrhagic events and identifying candidates for reperfusion therapies [3, 4]. Parallel to advances in imaging, the National Institutes of Health Stroke Scale (NIHSS) has been widely adopted as a standardized measure of stroke severity, correlating with infarct volume, large vessel occlusion likelihood, and functional outcomes [5, 6].

Studies have projected an increase in the number of strokes which will contribute to already overburdened healthcare systems [7]. A recent study at an academic institution has proposed a minimum NIHSS for initiating imaging as a means to reduce healthcare system burden and costs [8]. In addition to the increasing number of stroke imaging, another challenge for radiologists is whether NIHSS scores are available at the time of emergent imaging and how this information (or lack thereof) impacts the interpretation and clinical decision-making. A previous study explored the integration of NIHSS with imaging workflow at a major academic center [8]. However, this remains underexplored in the community hospital setting. Moreover, many institutions have utilized tele-neurology to address this increasing demand, particularly within the community hospital setting with a shortage of on-site neurology teams [9]. One might speculate whether this could influence the clinical data available and communicated for imaging interpretation.

This study evaluates NIHSS availability at the time of imaging for code stroke activations in a community hospital. We sought to determine correlations between NIHSS documentation, imaging positivity, and clinical outcomes, while comparing our findings with prior literature from academic centers.

## Materials and Methods

A retrospective cohort study was conducted at a community hospital designated as a Level I stroke center. All patients who triggered a code stroke activation between October 1, 2021 and September 30, 2023, when benchmarks were last adapted, were included. Code stroke activation was based on acute neurological symptoms consistent with stroke within an appropriate time window, in alignment with national guidelines [10]. Following activation, patients underwent emergent non-contrast head CT and CT angiography (CTA) of the head and neck, which was immediately reviewed by the on-call radiologist. An MRI of the brain was subsequently performed.

Electronic medical records were reviewed to collect data including patient demographics (sex), vascular risk factors (hypertension, hyperlipidemia, diabetes), timing variables (door-to-CT and door-to-NIHSS times), NIHSS scores (presence, timing, score value, provider type [in-house vs. tele-neurology]), imaging findings (ischemia, hemorrhage, large vessel occlusion), treatments received (tPA, thrombectomy), and final neurologic impression prior to discharge (stroke, TIA, or other). Imaging was considered positive if there was evidence of ischemic infarction, intracerebral hemorrhage, or major vascular occlusion on initial CT/CTA or subsequent MRI.

Descriptive statistical metrics were calculated for baseline characteristics and outcomes. Continuous variables such as door-to-CT and door-to-NIHSS times were compared using paired two-tailed *t*-tests in which a *p*-value of <0.001 was considered statistically significant. Differences in mean NIHSS scores between groups (positive vs. negative imaging findings) were compared using independent two-tailed *t*-tests.

NIHSS availability at the time of initial imaging interpretation was collected and an odds ratio (OR) with a 95% confidence interval was calculated. Using this data, a receiver operating characteristic (ROC) curve was generated and an area under the curve (AUC) was calculated. Statistical analyses were performed using Microsoft Excel v16.83 and v16.95.1.

## Results

**Figure 1:**
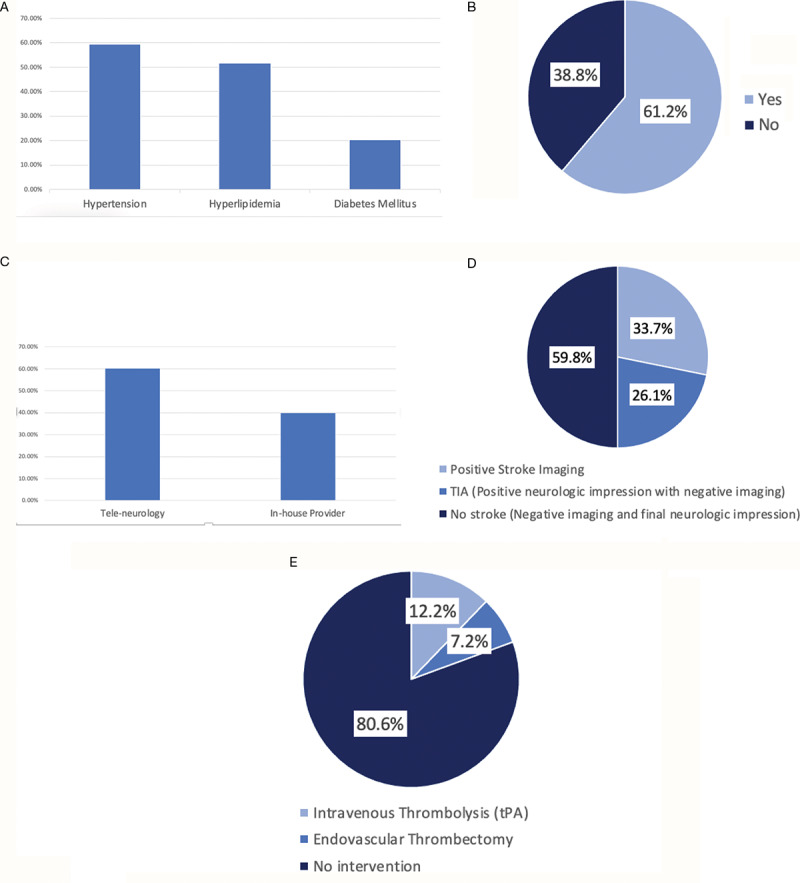
Percentile distribution of risk factors for the total stroke codes **(A)**, percentage of NIHSS scores available at time of initial imaging **(B)**, percentage distribution of NIHSS screening assessment by personel **(C)**, total stroke case breakdown by imaging and neurologic impression **(D)**, clinical intervention of the total code stroke cases **(E)**.

A total of 291 patients activated code stroke and underwent emergent imaging during the study period. The cohort included 151 females and 140 males. Common vascular risk factors included hypertension (59.5%), hyperlipidemia (51.9%), and diabetes mellitus (20.3%) (Figure 1A).

An NIHSS score was eventually documented for 98.6% (287/291) of patients. However, NIHSS documentation prior to imaging was available in only 61.2% (178/291) of the cases (Figure 1B). Overall, NIHSS screening was performed by an in-house provider in 39.9% of the cases and by tele-neurology in 60.1% of the cases (Figure 1C).

The median door-to-CT time was 12 minutes (median 12; mode 9; range 144 minutes), while the median door-to-NIHSS time was 29 minutes (median 29; mode 6; range 252 minutes). A two-tailed *t*-test was performed, where *p* < 0.001 and *P*(*T* ≤ *t*) = 3.3575e-14.

Positive stroke findings were present in 33.7% (98/291) of patients. An additional 26.1% (76/291) were positive by final neurologic impression of TIA, resulting in an overall stroke positivity (clinical and imaging) of 59.8% (174/291) (Figure 1D).

Among patients with pre-imaging NIHSS scores, 36.5% had positive imaging findings compared to 28.0% in those without NIHSS prior to imaging. Patients with NIHSS documented prior to imaging had a higher stroke positivity rate with an OR of 1.45 (95% CI 0.86–2.42). Though suggestive, this difference was not statistically significant.

**Figure 2:**
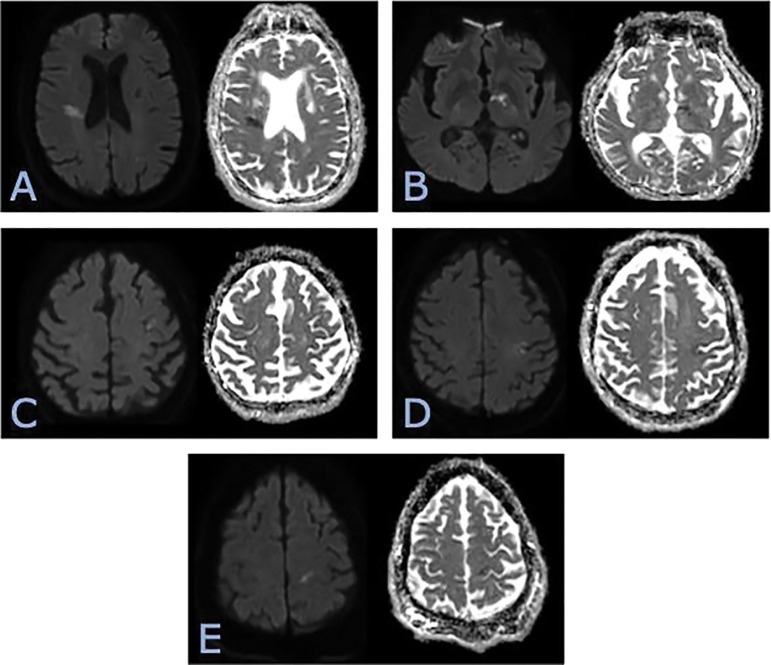
Diffusion weighted MRI images in the axial plane of multiple patients with small acute infarcts with NIHSS of zero including - **(A)** right corona radiata, **(B)** left basal ganglia, **(C)** posterior left frontal lobe cortex, **(D)** posterior left frontal lobe cortex, **(E)** posterior left frontal lobe cortex.

The overall NIHSS score mean was 6.3 (median 4; mode 0, range 30). The mean NIHSS in patients with imaging-positive stroke was 9.1 (median 6.5; mode 2, range 28). The mean NIHSS in patients with negative imaging was 3.7 (median 3; mode 0, range 24). Notably, eight patients (2.7%) demonstrated positive imaging findings despite an NIHSS score of 0. These cases often involved small infarcts, including within the cortex, basal ganglia, and corona radiata (Figures 2A–E).

**Figure 3:**
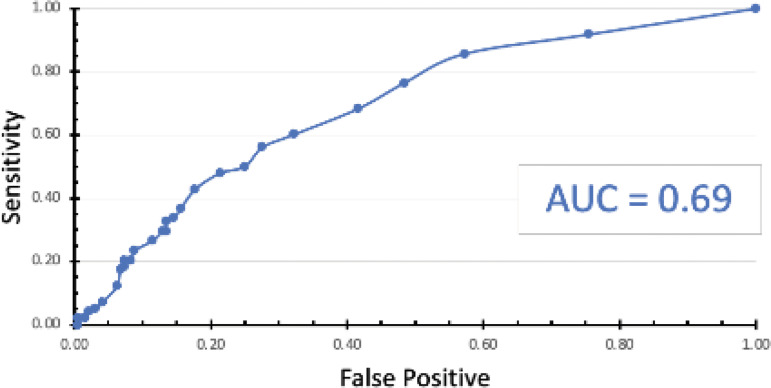
Receiver operating curve for NIHSS predicting imaging positivity.

The ROC curve analysis for NIHSS predicting imaging positivity yielded an AUC of 0.69, reflecting moderate discriminative ability (Figure 3). No clear NIHSS threshold achieved both high sensitivity and specificity simultaneously.

Treatment intervention demonstrated that 12.2% (36/291) received intravenous thrombolysis (tPA) while 7.2% (21/291) underwent endovascular thrombectomy for large vessel occlusion (Figure 1E).

## Discussion

Our study highlights key insights into the integration of clinical stroke severity assessment via NIHSS with emergent imaging workflows at a community Level I stroke center. In particular, it identifies gaps between imaging acquisition and clinical information availability, especially the lag in NIHSS documentation relative to CT/CTA acquisition. These findings have important implications for radiologists, neurologists, and stroke program optimization.

We found that NIHSS documentation was missing or delayed relative to initial image interpretation in 38.8% of code stroke cases. This is consistent with prior studies reporting incomplete NIHSS availability during stroke imaging workups at academic centers [8]. Moreover, this aligns with a national analysis which demonstrated a significant minority of stroke cases with incomplete NIHSS documentation during hospital admissions [11].

The median door-to-CT time in our study was 12 minutes—a metric consistent with American Heart Association (AHA) targets—while the door-to-NIHSS time was significantly longer at 29 minutes [10]. The difference was statistically significant utilizing a two-tailed *t*-test, indicating that imaging typically occurred well before NIHSS documentation. This gap reflects real-world priorities: imaging acquisition is prioritized to minimize delays to thrombolysis or thrombectomy. However, the lack of NIHSS availability at the time of imaging interpretation poses challenges for radiologists, particularly in cases with minimal or equivocal image findings. Prior studies by Sarraj et al. and Albers et al. have emphasized the importance of integrating clinical information with imaging to maximize diagnostic accuracy and guide treatment decisions [12, 13]. Furthermore, Hsia et al. showed that structured communication between stroke teams and radiologists improves imaging interpretation and acute stroke care efficiency [14]. As such, enhancing workflows to ensure earlier NIHSS availability, perhaps through the implementation of stroke-specific templates to speed up NIHSS entry and visibility or formalizing communication of the NIHSS score, either by including it within the indication or the uploaded imaging documents, could support radiologists in acute stroke scenarios.

Our data affirm that higher NIHSS scores correlate with a greater likelihood of imaging-positive stroke. Patients with imaging-confirmed strokes had a mean NIHSS of 9.1, versus 3.7 in those without stroke. This trend aligns with previous studies showing NIHSS as a strong predictor of large vessel occlusion and infarct size [15, 16]. However, we also found that eight patients (2.7% of the total cases) had NIHSS scores of 0 yet showed clear infarcts on imaging—a finding more common than the 0.76% rate reported by Martin-Schild et al. [17]. Similar concerns have been raised by studies documenting that small posterior circulation strokes or cortical strokes can present with deceptively normal NIHSS scores [17, 18]. Similarly, multiple cases demonstrated small infarcts within the corona radiata (Figure 2A), basal ganglia (Figure 2B), and cortex (Figures 2C–E). This is consistent with previous studies emphasizing the danger of relying solely on NIHSS to triage imaging decisions and that NIHSS scores should not exclude stroke from the differential, especially when clinical suspicion persists.

Isikbay et al. proposed an NIHSS cutoff of 3 to achieve near-100% sensitivity for detecting imaging-positive strokes in their tertiary center cohort [8]. In contrast, our data suggest that no safe cutoff exists in the community setting since patients with NIHSS less than 3 had imaging-positive strokes. This discrepancy may be attributed to differences in patient factors (age, additional morbidities), and physical exam variability (examiner bias, subjective nature of some findings). The ROC AUC for NIHSS predicting imaging positivity in our study was 0.69, similar to previous studies [19]. This suggests the moderate predictive power of NIHSS in community hospital populations but highlights that NIHSS alone is insufficient for ruling in or ruling out stroke.

A major trend we observed was the utilization of remote tele-neurology services. Tele-neurology performed 60.2% of the NIHSS assessments in our study. Prior work has shown that tele-neurology assessments demonstrate high diagnostic accuracy and are both reliable and comparable to in-person evaluations for NIHSS scoring and treatment decisions [20, 21]. However, minor delays in NIHSS documentation related to teleconsultation logistics could contribute to the observed lag in clinical information relative to imaging in our study.

This study’s strengths include its real-world focus in a community hospital setting and its comprehensive analysis of both imaging and clinical outcomes across a two-year cohort. However, several limitations merit discussion. This study was performed at a single center and the findings may not be generalized to other community hospitals. Additionally, NIHSS time documentation is human-dependent and may be subject to recall bias as compared to imaging timing, which is computerized and automatic. Moreover, our study did not assess long-term functional recovery or modified Rankin scale scores.

Future multicenter studies examining NIHSS timing, imaging findings, and outcomes could further validate these observations across diverse practice environments.

## Conclusion

In this retrospective study of code stroke activations at a community hospital, we found that while emergent imaging is performed rapidly and consistently with national benchmarks, NIHSS documentation often lags behind imaging acquisition. Despite this, higher NIHSS scores correlated with an increased likelihood of imaging-positive stroke, reinforcing the prognostic value of NIHSS.

However, a notable proportion of patients with NIHSS scores of 0 still harbored acute infarcts. Moreover, this contrasts with similar research at academic institutions, which has suggested NIHSS cutoffs to address overburdened healthcare systems. Instead, our data highlight the importance of clinical judgment and suggest that a low threshold for imaging must prevail even when NIHSS is minimal.

Community stroke centers, increasingly reliant on tele-neurology, must consider workflow optimizations to ensure early NIHSS documentation and timely communication to interpreting radiologists. Structured processes could enhance imaging interpretation, reporting quality, and ultimately, patient outcomes.

Future research should explore the impact of improved clinical-radiology communication strategies on stroke diagnosis accuracy and therapeutic efficiency in varied practice settings.

## References

[r1] Feigin VL, Owolabi MO. Pragmatic solutions to reduce the global burden of stroke: A world stroke organization perspective. Lancet Neurol. 2023; 22(12): 1160–1206. https://www.thelancet.com/journals/laneur/article/PIIS1474-4422(23)00277-6/fulltext. 37827183 10.1016/S1474-4422(23)00277-6 PMC10715732

[r2] Johnson W, Onuma O, Owolabi M, Sachdev S. Stroke: A global response is needed. Bull World Health Organ. 2016; 94(9): 634–634A. https://pubmed.ncbi.nlm.nih.gov/27708464/. 27708464 10.2471/BLT.16.181636 PMC5034645

[r3] Birenbaum D, Bancroft L, Felsberg G. Imaging in acute stroke. West J Emerg Med. 2011; 12(1): 67–76. https://pubmed.ncbi.nlm.nih.gov/21694755/. 21694755 PMC3088377

[r4] Powers W, Rabinstein A, Ackerson T, Adeoye O, Bambakidis N, Becker K, et al. Guidelines for the early management of patients with acute ischemic stroke: 2019 update to the 2018 guidelines for the early management of acute ischemic stroke: A guideline for healthcare professionals from the American Heart Association/American Stroke Association. Stroke. 2019; 50(12): e344–e418. https://www.ahajournals.org/doi/10.1161/STR.0000000000000211. 31662037 10.1161/STR.0000000000000211

[r5] Meyer B, Lyden P. The modified national institutes of health stroke scale: Its time has come. Int J Stroke. 2010; 5(4): 267–273. https://pubmed.ncbi.nlm.nih.gov/19689755/. 10.1111/j.1747-4949.2009.00294.x PMC2729912 19689755

[r6] Saver J. Time is brain—quantified. Stroke. 2006; 37(1): 263–266. https://pubmed.ncbi.nlm.nih.gov/16339467/. 16339467 10.1161/01.STR.0000196957.55928.ab

[r7] Cheng Y, Yongqi L, Hujuan S, Cheng M, Zhang B, Liu X, et al. Projections of the stroke burden at the global, regional, and national levels up to 2050 based on the global burden of disease study 2021. J Am Heart Assoc. 2024; 13(23): e036142. https://pmc.ncbi.nlm.nih.gov/articles/PMC11681572. 39575720 10.1161/JAHA.124.036142 PMC11681572

[r8] Isikbay J, Talbott J. Evaluating the utilization and utility of the NIH stroke scale in the workup of stroke with imaging. In: American Society of Neuroradiology Annual Meeting Abstracts; 2023.

[r9] Patel UK, Malik P, DeMasi M, Lunagariya A, Jani VB. Multidisciplinary approach and outcomes of tele-neurology: A review. Cureus. 2019; 811(4): e4410. https://pmc.ncbi.nlm.nih.gov/articles/PMC6561521/. 10.7759/cureus.4410 PMC6561521 31205830

[r10] American Heart Association/American Stroke Association. Target: Stroke Phase III Manual. 2019.

[r11] Reeves MJ, Smith EE, Fonarow GC, Zhao X, Thompson M, Peterson E, et al. Variation and trends in documentation of NIH stroke scale scores in U.S. hospitals. Circ Cardiovasc Qual Outcomes. 2015; 8(6 Suppl 3):S90–S98. https://www.ahajournals.org/doi/10.1161/CIRCOUTCOMES.115.001775. 26515215 10.1161/CIRCOUTCOMES.115.001775

[r12] Sarraj A, Hassan A, Grotta J, Blackburn S, Day A, Abraham M, et al. Early infarct growth rate correlation with endovascular thrombectomy clinical outcomes: Analysis from the select study. Stroke. 2021; 52(1): 57–69. https://pubmed.ncbi.nlm.nih.gov/33280550/. 33280550 10.1161/STROKEAHA.120.030912

[r13] Albers GS, Marks MP, Kemp S, Christensen S, Tsai JP, Oretega-Guitierrez S, et al. Thrombectomy for stroke at 6 to 16 hours with selection by perfusion imaging. N Engl J Med. 2018; 378(8): 708–718. https://www.nejm.org/doi/full/10.1056/NEJMoa1713973. 29364767 10.1056/NEJMoa1713973 PMC6590673

[r14] Hsia AW, Castle A, Wing JJ, Edwards D, Brown N, Higgins T, et al. Understanding reasons for delay in seeking acute stroke care in an underserved urban population. Stroke. 2011; 42(6): 1697–1701. https://pubmed.ncbi.nlm.nih.gov/21546471/. 21546471 10.1161/STROKEAHA.110.604736 PMC3130551

[r15] Smith E, Kent D, Bulsara K, Leung L, Lichtman J, Reeves M, et al. Accuracy of prediction instruments for diagnosing large vessel occlusion in suspected stroke: A systematic review for the 2018 guidelines for the early management of patients with acute ischemic stroke. Stroke. 2018; 49(3): e111–e122. https://pubmed.ncbi.nlm.nih.gov/29367333/. 29367333 10.1161/STR.0000000000000160

[r16] Heldner M, Hsieh K, Broeg-Morvay A, Mordasini P, Buhlmann M, Jung S, et al. Clinical prediction of large vessel occlusion in anterior circulation stroke: mission impossible? J Neurol. 2016; 263(8): 1633–1640. https://pubmed.ncbi.nlm.nih.gov/27272907/. 27272907 10.1007/s00415-016-8180-6

[r17] Martin-Schild S, Albright K, Tanksley J, Pandav V, Jones E, Grotta J, et al. Zero on the NIHSS does not equal the absence of stroke. Ann Emerg Med. 2011; 57(1): 42–45. https://pubmed.ncbi.nlm.nih.gov/20828876/. 20828876 10.1016/j.annemergmed.2010.06.564 PMC3426834

[r18] Eskioglou E, Huchmandzadeh M, Amiguet M, Michel P. National institute of health stroke scale zero strokes. Stroke. 2018; 49(12): 3057–3059. https://www.ahajournals.org/doi/full/10.1161/STROKEAHA.118.022517. 30571424 10.1161/STROKEAHA.118.022517

[r19] Yaghi S, Herber C, Willey J, Andrews H, Boehme A, Marshall R, et al. Itemized NIHSS subsets predict positive MRI strokes in patients with mild deficits. J Neurol Sci. 2015; 358 (1–2): 221–225. https://pmc.ncbi.nlm.nih.gov/articles/PMC5541946/. 26375623 10.1016/j.jns.2015.08.1548 PMC5541946

[r20] Demaerschalk B, Raman R, Ernstrom K, Meyer B. Efficacy of telemedicine for stroke: Pooled analysis of the stroke team remote evaluation using a digital observation camera (STRoKE DOC) and stroke doc Arizona telestroke trials. Telemed J E Health. 2012; 18(3): 230–237. https://pubmed.ncbi.nlm.nih.gov/22400970/. 22400970 10.1089/tmj.2011.0116 PMC3317394

[r21] Silva G, Farrell S, Shandra E, Viswanathan A, Schwamm L. The status of telestroke in the United States: A survey of currently active stroke telemedicine programs. Stroke. 2012; 43(8): 2078–2085. https://www.ahajournals.org/doi/10.1161/strokeaha.111.645861. 22700532 10.1161/STROKEAHA.111.645861

